# Prognosis of Transthyretin Cardiac Amyloidosis Without Heart Failure Symptoms

**DOI:** 10.1016/j.jaccao.2022.07.007

**Published:** 2022-11-15

**Authors:** Esther Gonzalez-Lopez, Luis Escobar-Lopez, Laura Obici, Giulia Saturi, Mélanie Bezard, Sunil E. Saith, Omar F. AbouEzzeddine, Roberta Mussinelli, Christian Gagliardi, Mounira Kharoubi, Jan M. Griffin, Angela Dispenzieri, Silvia Vilches, Stefano Perlini, Simone Longhi, Silvia Oghina, Adrian Rivas, Martha Grogan, Mathew S. Maurer, Thibaud Damy, Giovanni Palladini, Claudio Rapezzi, Pablo Garcia-Pavia

**Affiliations:** a Heart Failure and Inherited Cardiac Diseases Unit, Department of Cardiology, Hospital Universitario Puerta de Hierro, IDIPHISA, CIBERCV, Madrid, Spain; b Amyloidosis Research and Treatment Center, Fondazione IRCCS Policlinico S. Matteo, Pavia, Italy; c Cardiology, Department of Diagnostic, Experimental and Specialty Medicine, University of Bologna and S. Orsola-Malpighi Hospital, Bologna, Italy; d Referral Center for Cardiac Amyloidosis, GRC Amyloid Research Institute, Department of Cardiology, Center Hospitalier Universitaire Henri Mondor, DHU-ATVB Créteil, France; e Inserm U955, Université Paris-Est Créteil, Créteil, France; f Division of Cardiology, Department of Medicine, Columbia University Irving Medical Center, New York New York, USA; g Department of Cardiovascular Diseases, Mayo Clinic, Rochester, Minnesota, USA; h Emergency Department, Fondazione IRCCS Policlinico S. Matteo, Internal Medicine Department, University of Pavia, Pavia, Italy; i Cardiovascular Center, University of Ferrara, Ferrara, Italy; j Maria Cecilia Hospital, GVM Care & Research, Cotignola, Italy; k Universidad Francisco de Vitoria, Pozuelo de Alarcón, Spain; l Centro Nacional de Investigaciones Cardiovasculares, Madrid, Spain

**Keywords:** cardiac amyloidosis, early stages, heart failure, stabilizers, transthyretin, AF, atrial fibrillation, ATTR-CM, transthyretin amyloid cardiomyopathy, ATTR-v, variant transthyretin amyloidosis, ATTR-wt, wild-type transthyretin amyloidosis, CV, cardiovascular, eGFR, estimated glomerular filtration rate, HF, heart failure, NAC, National Amyloid Center, NT-proBNP, N-terminal pro–brain natriuretic peptide, NYHA, New York Heart Association, SHR, subdistribution HR, TTR, transthyretin

## Abstract

**Background:**

Transthyretin amyloid cardiomyopathy (ATTR-CM) is increasingly recognized as a treatable cause of heart failure (HF). Advances in diagnosis and therapy have increased the number of patients diagnosed at early stages, but prognostic data on patients without HF symptoms are lacking. Moreover, it is unknown whether asymptomatic patients benefit from early initiation of transthyretin (TTR) stabilizers.

**Objectives:**

The aim of this study was to describe the natural history and prognosis of ATTR-CM in patients without HF symptoms.

**Methods:**

Clinical characteristics and outcomes of patients with ATTR-CM without HF symptoms were retrospectively collected at 6 international amyloidosis centers.

**Results:**

A total of 118 patients (78.8% men, median age 66 years [IQR: 53.8-75 years], 68 [57.6%] with variant transthyretin amyloidosis, mean left ventricular ejection fraction 60.5% ± 9.9%, mean left ventricular wall thickness 15.4 ± 3.1 mm, and 53 [45%] treated with TTR stabilizers at baseline or during follow-up) were included. During a median follow-up period of 3.7 years (IQR: 1-6 years), 38 patients developed HF symptoms (23 New York Heart Association functional class II and 14 functional class III or IV), 32 died, and 2 required cardiac transplantation. Additionally, 20 patients received pacemakers, 13 developed AF, and 1 had a stroke. Overall survival was 96.5% (95% CI: 91%-99%), 90.4% (95% CI: 82%-95%), and 82% (95% CI: 71%-89%) at 1, 3, and 5 years, respectively. Treatment with TTR stabilizers was associated with improved survival (HR: 0.31; 95% CI: 0.12-0.82; *P* = 0.019) and remained significant after adjusting for sex, age, ATTR-CM type, and estimated glomerular filtration rate (HR: 0.18; 95% CI: 0.06-0.55; *P* = 0.002).

**Conclusions:**

After a median follow-up period of 3.7 years, 1 in 3 patients with asymptomatic ATTR-CM developed HF symptoms, and nearly as many died or required cardiac transplantation. Treatment with TTR stabilizers was associated with improved prognosis.

Transthyretin amyloid cardiomyopathy (ATTR-CM) is a progressive and fatal cardiomyopathy caused by the extracellular deposition of transthyretin (TTR) amyloid fibrils in the heart, either in its variant or wild-type form.^1^

Advances in noninvasive imaging techniques as well in the definition of noninvasive diagnostic criteria and the availability of new treatments have led to increased recognition of ATTR-CM, and patients are now diagnosed at earlier disease stages.^2^, ^3^, ^4^, ^5^, ^6^

Several studies have demonstrated the important contribution of ATTR-CM to heart failure (HF) in elderly patients.^7^, ^8^, ^9^ Once HF is present, ATTR-CM is associated with a life expectancy of only 2.5 to 3.5 years if left untreated, but no data are available on the prognosis and natural history of patients with ATTR-CM diagnosed at earlier stages.^10^, ^11^, ^12^ As a result, delineating prognosis in this growing group of patients is problematic.

Tafamidis, a TTR stabilizer that attenuates TTR dissociation and thereby slows amyloid fibril formation, was recently shown to reduce mortality and cardiac hospitalizations in patients with symptomatic HF with ATTR-CM.^13^ Additional studies evaluating other stabilizers or TTR gene-silencing agents are ongoing, but again these studies include only patients with ATTR-CM and clinical HF.^14^ As a consequence, it is unknown whether patients with ATTR-CM without HF symptoms could benefit from early initiation of specific therapies.

Here, we sought to characterize patients with ATTR-CM without HF symptoms and to describe their outcomes and prognosis. We also explored the effects of disease-modifying therapies in the subgroup of patients already taking these drugs despite the absence of HF symptoms.

## Methods

The present study conforms to the principles of the Declaration of Helsinki, and all authors guarantee the integrity of data from their respective institutions. Approval from a local ethics committee or internal review board was obtained at each participating center.

### Cohort composition

This was a multicenter, longitudinal cohort study comprising consecutive patients with ATTR-CM without HF symptoms evaluated at 4 European (Hospital Universitario Puerta de Hierro Majadahonda, Spain; Fondazione IRCCS Policlinico S. Matteo, Italy; University of Bologna and S. Orsola-Malpighi Hospital, Italy; and University Hospital Henri Mondor, France) and 2 U.S. (Columbia University Irving Medical Center, New York, New York; and Mayo Clinic, Rochester, Minnesota) amyloidosis centers.

Adult patients with variant transthyretin amyloidosis (ATTR-v) or wild-type transthyretin amyloidosis (ATTR-wt) with ATTR-CM and without HF symptoms or histories of HF symptoms at initial evaluation were retrospectively identified from existing databases. Clinical HF was considered on the basis of current guidelines, at a stage at which clinical symptoms are apparent and may be accompanied by signs.^15^ ATTR-CM was diagnosed by any of the following: 1) demonstration of TTR amyloid deposits on endomyocardial biopsy; 2) demonstration of TTR amyloid deposits on extracardiac biopsy and at least 1 of the following: a) increased left ventricular wall thickness (≥12 mm) on echocardiography, not explained by disturbances in loading conditions (ie, hypertension, aortic stenosis); b) cardiac magnetic resonance findings consistent with cardiac amyloidosis (ie, diffuse subendocardial or transmural late gadolinium enhancement with abnormal gadolinium kinetics); and c) cardiac uptake grade 2 or 3 on planar/single-photon emission computed tomographic ^99m^Tc-3,3-diphosphono-1,2-propanedicarboxylic acid/^99m^Tc-pyrophosphate/hydroxymethylene diphosphonate bone scintigraphy; and 3) cardiac uptake grade 2 or 3 on planar/single-photon emission computed tomographic ^99m^Tc-3,3-diphosphono-1,2-propanedicarboxylic acid/^99m^Tc-pyrophosphate/hydroxymethylene diphosphonate scintigraphy and no evidence of monoclonal protein in the presence of findings suggestive of amyloid on echocardiography or cardiac magnetic resonance.^3^

Patients were required to be in New York Heart Association (NYHA) functional class I and be free of HF symptoms at initial evaluation at participating centers. Additionally, patients were excluded if they were receiving or had received loop diuretic agents, had signs of HF at diagnosis (eg, elevated jugular venous pressure, pulmonary crackles, and/or peripheral edema), had prior HF hospitalizations or a prior N-terminal pro–brain natriuretic peptide (NT-proBNP) level ≥600 pg/mL (when available), as this was the cutoff value required for inclusion of symptomatic patients in the tafamidis clinical trial.^13^ In all cases, genetic testing confirmed the absence or presence of mutations in the TTR gene.

### Data collection

Demographic and clinical data at baseline evaluation were extracted from available hospital records. Age, type, and date of ATTR-CM diagnosis and symptoms leading to diagnosis were obtained. Presence of neurologic symptoms, history of embolism, hypertension, coronary artery or valve disease, atrial fibrillation (AF), and pacemaker implantation were also collected. Hypertension was defined on the basis of clinical history or use of at least 1 antihypertensive medication at presentation. Coronary artery disease was defined as a history of myocardial infarction or by the presence of at least 1 moderate coronary artery stenosis. Basal characteristics extracted also included estimated glomerular filtration rate (eGFR) (calculated using the Chronic Kidney Disease Epidemiology Collaboration formula), cardiac biomarkers, presence of conduction abnormalities on electrocardiography, and echocardiographic parameters obtained at first evaluation.

Time to follow-up began at the time of first evaluation at each center. Treatment with ATTR-specific therapies was recorded. Cardiovascular (CV) events including cardiac transplantation, HF admission, pacemaker implantation, AF appearance, stroke, and other thromboembolic events during follow-up were captured.

Information on patients’ status at last follow-up was obtained from medical records. Overall and CV mortality was defined as mortality due to any cause or to cardiac complications.

### Statistical analysis

Normality was assessed using the Shapiro-Wilk test. Normally distributed variables are expressed as mean ± SD, while non-normally distributed variables are reported as median (IQR). Categorical data are reported as frequencies and percentages and were compared using the chi-square test or Fisher exact test. Comparison of continuous variables between 2 independent groups was performed using the unpaired Student’s *t*-test or the Mann-Whitney *U* test. Survival was evaluated from baseline evaluation with Kaplan-Meier estimates with 95% CIs. *P* values of <0.05 were considered to indicate statistical significance.

Moreover, to evaluate whether stabilizer treatment was associated with delayed HF onset or increased survival in patients without HF, the effect of stabilizer treatment on time to death and on time to HF onset was analyzed with stabilizers as a time-dependent covariate using the Cox proportional hazards regression model in the time-to-death analysis and by Fine and Gray’s approach (considering death before developing HF as a competing risk event) in the time-to-HF analysis. In both analyses, sex, age, ATTR-CM type, and eGFR were considered as covariates. eGFR was imputed using the median value for patients who did not have this parameter available. Eight patients who developed HF and for whom HF onset date was unknown were excluded from the time-to-HF analysis but were included in the time-to-death analysis. HRs are shown for the Cox model and subdistribution HRs (SHRs) for Fine and Gray’s approach with 95% CIs. As death from any cause was considered a competing event in the analysis of time to HF, the cumulative incidence function was estimated in the corresponding curves and used to determine the cumulative incidence of HF at various time points. A sensitivity analysis based on 150 resamples through bootstrapping was performed for the time-to-HF analysis.

Statistical analysis was performed using Stata SE version 16 (StataCorp) and SPSS Statistics version 22 (IBM).

## Results

A total of 118 patients with ATTR-CM without HF symptoms (median age 66 years [IQR: 53.8-75 years] at diagnosis, 78.8% men) were included in the study. Clinical, echocardiographic, and electrocardiographic findings at baseline evaluation are shown in Table 1.

**Table 1:** Baseline Characteristics of the Total Cohort and According to ATTR-v and ATTR-wt

	Total (N = 118)	ATTR-v (n = 68)	ATTR-wt (n = 50)	*P* Value
Baseline characteristics				
Sex				0.036
Male	93 (78.8)	49 (72.1)	44 (88)	
Female	25 (21.2)	19 (28.0)	6 (12)	
Race (n = 116)		(n = 67)	(n = 49)	0.30
White	111 (95.7)	63 (94.0)	48 (98.0)	
Black	5 (4.3)	4 (6.0)	1 (2.0)	
Age at diagnosis, y	66 (53.8-75)	57 (49.8-66)	73.5 (69-80.5)	<0.001
ATTR-v genotype				
p.Val50Met (V30M)	21 (17.8)	21 (30.9)		
p.Val142Ile (V122I)	4 (3.4)	4 (5.9)		
p.Glu109Gln (E89Q)	13 (11.0)	13 (19.1)		
p.Glu109Lys (E89K)	1 (0.8)	1 (1.5)		
p.Thr80Ala (T60A)	1 (0.8)	1 (1.5)		
p.Ile88Leu (I68L)	5 (4.2)	5 (7.4)		
p.Thr69Ala (T49S)	4 (3.4)	4 (5.9)		
p.Phe84Leu (F64L)	4 (3.4)	4 (5.9)		
p.Ala56Pro (A36P)	2 (1.7)	2 (2.9)		
p.Thr79Lys (T59K)	2 (1.7)	2 (2.9)		
Other mutations	11 (9.3)	11 (16.2)		
Type of diagnosis (n = 118)				<0.001
Histological by EMB	37 (31.4)	28 (41.2)	9 (18.0)	
Histological extracardiac	30 (25.4)	23 (33.8)	7 (14.0)	
Noninvasive	51 (43.2)	17 (25)	34 (68.0)	
Era diagnosed (n = 118)				<0.001
Early (1993-1999)	8 (6.8)	8 (11.8)	0	
Middle (2000-2009)	21 (17.8)	18 (26.5)	3 (6.0)	
Late (2010-2019)	89 (75.4)	42 (61.8)	47 (94.0)	
Circumstances leading to diagnosis (n = 113)		(n = 67)	(n = 46)	<0.001
Differential diagnosis LVH	17 (15)	6 (9)	11 (23.9)	
Cardiac surveillance of ATTR-v	31 (27.4)	31 (46.3)	0	
ATTR-v familial screening	25 (22.1)	25 (37.3)	0	
Incidental by scintigraphy	17 (15)	3 (4.5)	14 (30.4)	
Conduction/rhythm abnormalities	8 (7.1)	1 (1.5)	7 (15.2)	
Incidental by histology	5 (4.4)	1 (1.5)	4 (8.7)	
Part of aortic stenosis workup	1 (0.9)	0	1 (2.2)	
Others	9 (8)	0	9 (19.6)	
Polyneuropathy	75 (63.6)	59 (86.8)	16 (32)	<0.001
History of embolism (n = 118)	2 (1.7)	2 (2.9)	0	0.22
History of atrial fibrillation	15 (12.7)	5 (7.4)	10 (20)	0.042
History of hypertension (n = 118)	41 (34.7)	14 (20.6)	27 (54)	<0.001
Coronary artery disease	12 (10.2)	5 (7.4)	7 (14)	0.24
Left-sided valve disease (moderate or greater)	7 (5.9)	1 (1.5)	6 (12.0)	0.017
Pacemaker	5 (4.2)	1 (1.5)	4 (8)	0.082
Chronic kidney disease	3 (2.5)	1 (1.5)	2 (4)	0.39
Baseline blood tests				
eGFR, mL/min/1.73 m^2^ (n = 104)	84.4 ± 26.5	94 ± 27.8	71.3 ± 18.1	<0.001
NT-proBNP, pg/mL (n = 74)	308.1 (164.5-480)	308 (178.8-486.3)	285.5 (163-432.3)	0.66
Elevated troponin levels (n = 62)	14 (22.6)	7 (22.6)	7 (22.6)	1.00
Baseline ECG				
AF or atrial flutter (n = 118)	6 (5)	0	6 (12)	0.009
Conduction abnormalities (n = 118)	53 (44.9)	32 (47.1)	21 (42.0)	0.47
First-degree atrioventricular block	16 (30.6)	12 (17.6)	4 (8.0)	
LBBB	7 (5.9)	3 (4.4)	4 (8.0)	
RBBB	12 (10.2)	5 (7.4)	7 (14.0)	
Incomplete bundle branch block	18 (15.2)	12 (17.6)	6 (12.0)	
Baseline echocardiography				
Interventricular septal wall thickness, mm (n = 117)	15.4 ± 3.1	15.3 ± 3.6	15.4 ± 2.3	0.97
Posterior wall thickness, mm (n = 115)	13.9 ± 2.8	14.0 ± 2.8	13.8 ± 2.8	0.77
End-diastolic left ventricular diameter, mm (n = 111)	44.4 ± 6.4	44.1 ± 6.8	44.8 ± 5.8	0.58
Left ventricular ejection fraction, % (n = 117)	60.5 ± 9.9	61.6 ± 10.6	59.2 ± 8.9	0.18
Global longitudinal strain, % (n = 47)	−15.8 ± 2.9	−15.6 ± 3.7	−16.0 ± 1.5	0.64
Left atrial diameter, mm (n = 95)	40.2 ± 6.3	39.5 ± 6.4	41.7 ± 5.9	0.096
Cardiac uptake in bone scintigraphy (n = 61)		(n = 25)	(n = 36)	0.36
Grade 0	2 (3.3)	2 (8)	0	
Grade 1	4 (6.6)	2 (8)	2 (5.6)	
Grade 2	23 (37.7)	9 (36)	14 (38.9)	
Grade 3	32 (52.5)	12 (48)	20 (55.6)	
ATTR-specific treatment at baseline^a^				
TTR stabilizer	19 (37.3)	13 (36.1)	6 (40)	0.79
TTR silencers	1 (8.3)	1 (8.3)	0	

Values are n (%), median (IQR), or mean ± SD.

AF = atrial fibrillation; ATTR = transthyretin amyloidosis; ATTR-v = variant transthyretin amyloidosis; ATTR-wt = wild-type transthyretin amyloidosis; ECG = electrocardiography; eGFR = estimated glomerular filtration rate; EMB = endomyocardial biopsy; LBBB = left bundle branch block; LVH = left ventricular hypertrophy; NT-proBNP = N-terminal pro–brain natriuretic peptide; RBBB = right bundle branch block; TTR = transthyretin.

a At baseline or started during the first 6 months after initial evaluation.

Sixty-eight patients (57.6%) had ATTR-v and 50 (42.4%) had ATTR-wt. Not unexpectedly, patients with ATTR-wt were significantly older than patients with ATTR-v at the time of diagnosis (73.5 years [IQR: 69-80.5 years] vs 57 years [IQR: 49.8-65.5 years]; *P* < 0.001). The majority of patients had been diagnosed invasively (n = 67 [56.8%]), but patients with ATTR-wt were more frequently diagnosed noninvasively (68% vs 25%; *P* < 0.001). Family screening and cardiac surveillance of patients with ATTR-v with polyneuropathy were the main causes leading to diagnosis of ATTR-CM in the ATTR-v population. Of note, 17 patients (15%) were incidentally diagnosed by a positive bone scan requested for noncardiac reasons and 5 patients (4.4%) following the incidental detection of TTR amyloid on biopsy (Table 1).

At baseline evaluation, 15 patients (12.7%) had histories of AF, 5 (4.2%) had permanent pacemakers, and 2 (1.7%) had histories of stroke or systemic embolism. Histories of AF, hypertension, and left-sided valve disease were significantly higher in patients with ATTR-wt (Table 1).

Median NT-proBNP was 308.1 pg/mL (IQR: 164.5-480 pg/mL), and 14 of the 62 patients (22.6%) with available troponin values exhibited raised levels. Electrocardiographic conduction abnormalities were found in 53 patients (44.9%). Patients showed moderately increased left ventricular wall thickness (mean interventricular septal thickness 15.4 ± 3.1 mm, mean posterior wall thickness 13.9 ± 2.8 mm) and preserved systolic function (mean left ventricular ejection fraction 60.5% ± 9.9%) but impaired global longitudinal strain (−15.8% ± −2.9%) (Table 1).

### Progression and survival data

**Figure 1:**
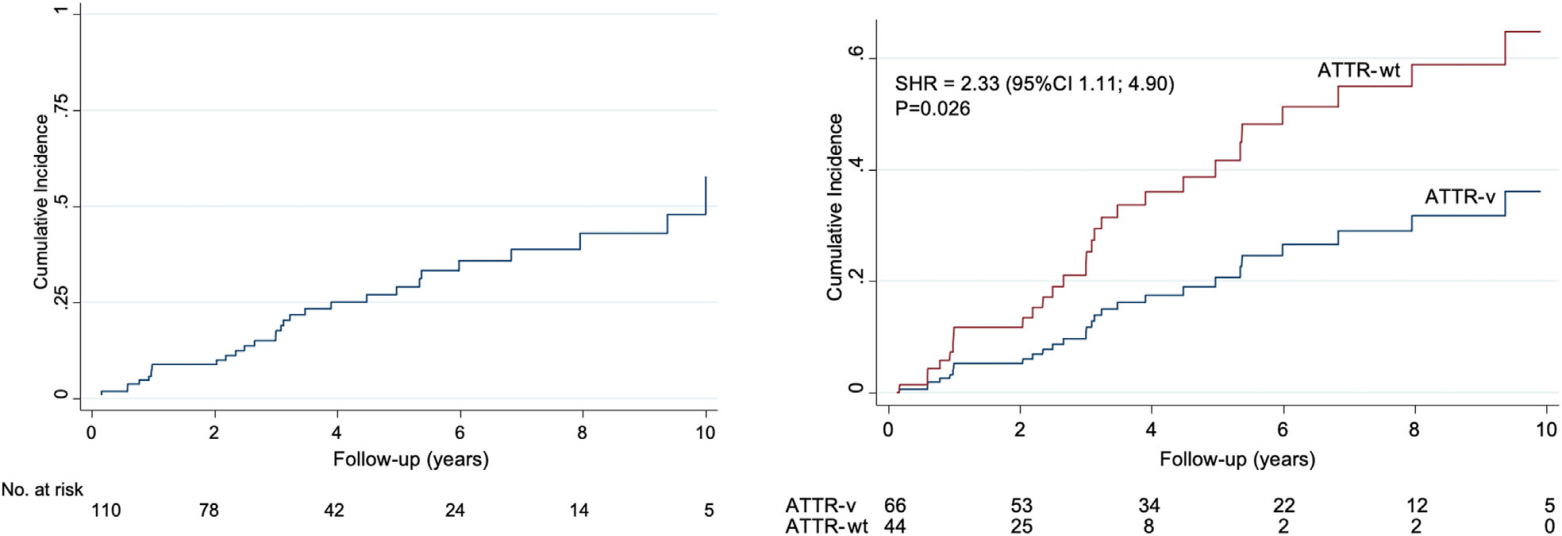
Heart Failure Development During Follow-Up Heart failure development in the entire cohort **(left)** and according to transthyretin amyloid cardiomyopathy type **(right)** (N = 110). ATTR-v = variant transthyretin amyloidosis; ATTR-wt = wild-type transthyretin amyloidosis; SHR = subdistribution HR.

During a median follow-up period of 3.7 years (IQR: 1-6 years), 38 patients developed clinical HF (defined as having been hospitalized for HF or progression to NYHA functional class ≥ II requiring diuretic agents), and 2 patients underwent cardiac transplantation. Median time from the first evaluation at each participant center to developing HF symptoms in the 30 individuals who developed HF and had HF onset date available was 3.05 years (IQR: 0.99-5.34), and the cumulative incidence of HF onset at 1, 3, and 5 years was 8% (95% CI: 4%-14%), 15% (95% CI: 9%-23%), and 27% (95% CI: 18%-37%), respectively (Figure 1). The respective cumulative incidences of HF in patients with ATTR-v vs ATTR-wt were as follows: at 1 year, 3.2% (95% CI: 1%-10%) vs 15.0% (95% CI: 6%-28%); at 3 years, 8.5% (95% CI: 3%-17%) vs 27% (95% CI: 13%-44%); and at 5 years, 20% (95% CI: 11%-32%) vs 47% (95% CI: 20%-70%).

At last follow-up, 77 patients remained in NYHA functional class I, while 37 had progressed to NYHA functional class ≥II, including 4 patients who were in NYHA functional class IV (Table 2). NYHA functional class at last follow-up was not available in 4 patients (3.4%). Median NT-proBNP at last follow-up was 459 pg/mL (IQR: 198.5-1,071.5 pg/mL), mean interventricular left ventricular wall thickness was 16.1 ± 3.2 mm, and left ventricular ejection fraction remained within the normal range (58.6% ± 10.5%) (Table 2).

**Table 2:** Follow-Up Characteristics of the Cohort and in ATTR-v and ATTR-wt

	Total (N = 118)	ATTR-v (n = 68)	ATTR-wt (n = 50)
Median follow-up, y	3 (1-6)	5 (2.8-7)	2 (1-4)
NYHA functional class at last follow-up (n = 114)			
I	77	46	31
II	23	10	13
III	10	7	3
IV	4	2	2
Initiation of ATTR-specific therapy during follow-up			
Initiation during first year of follow-up			
TTR stabilizer	32	24	8
TTR silencers	2	2	0
Blood test at last follow-up			
eGFR, mL/min/1.73 m^2^ (n = 84)	73.7 ± 24.7	78.5 ± 28.7	68.0 ± 17.7
NT-proBNP, pg/mL (n = 65)	459 (198.5-1,071.5)	506 (212-1,050.8)	414 (200-1,064)
ECG at last follow-up			
Sinus rhythm (n = 99)	73	43	30
Conduction abnormalities (n = 99)	35	18	17
First degree AV block	2	1	1
LBBB	6	3	3
RBBB	15	7	8
Incomplete bundle branch block	12	7	5
Echocardiography at last follow-up			
Interventricular septal wall thickness, mm (n = 103)	16.1 ± 3.2	16.0 ± 3.4	16.3 ± 2.9
Posterior wall thickness, mm (n = 95)	14.7 ± 2.9	14.3 ± 3.0	15.2 ± 2.6
End-diastolic left ventricular diameter, mm (n = 77)	44.7 ± 5.2	44.8 ± 4.8	44.5 ± 5.8
LVEF, % (n = 105)	58.6 ± 10.5	58.4 ± 11.6	58.9 ± 8.6
Global longitudinal strain, % (n = 45)	−15.1 ± 2.9	−15.2 ± 3.2	−15.0 ± 2.5
Left atrial diameter, mm (n = 50)	39.8 ± 5.4	40.1 ± 6.1	39.4 ± 4.3
Events			
AF during follow-up (n = 106)	13	5	8
Pacemaker implantation (n = 112)	20	15	5
Indications (n = 12)			
AV block	10	7	3
AV node ablation	1	0	1
Sinus node dysfunction	1	1	0
Stroke (n = 111)	1	1	0
HF during follow-up	38	20	18
Cardiac transplantation	2	2	0
Mortality	32	25	7
Causes of death			
Sudden cardiac death	4	3	1
Heart failure death	2	2	0
Other causes of CV death	2	2	0
Non-CV deaths	11	11	0
Not available	13	7	6

Values are median (IQR), n, or mean ± SD.

AV = atrioventricular; CV = cardiovascular; HF = heart failure; LVEF = left ventricular ejection fraction; NYHA = New York Heart Association; other abbreviations as in Table 1.

**Figure 2:**
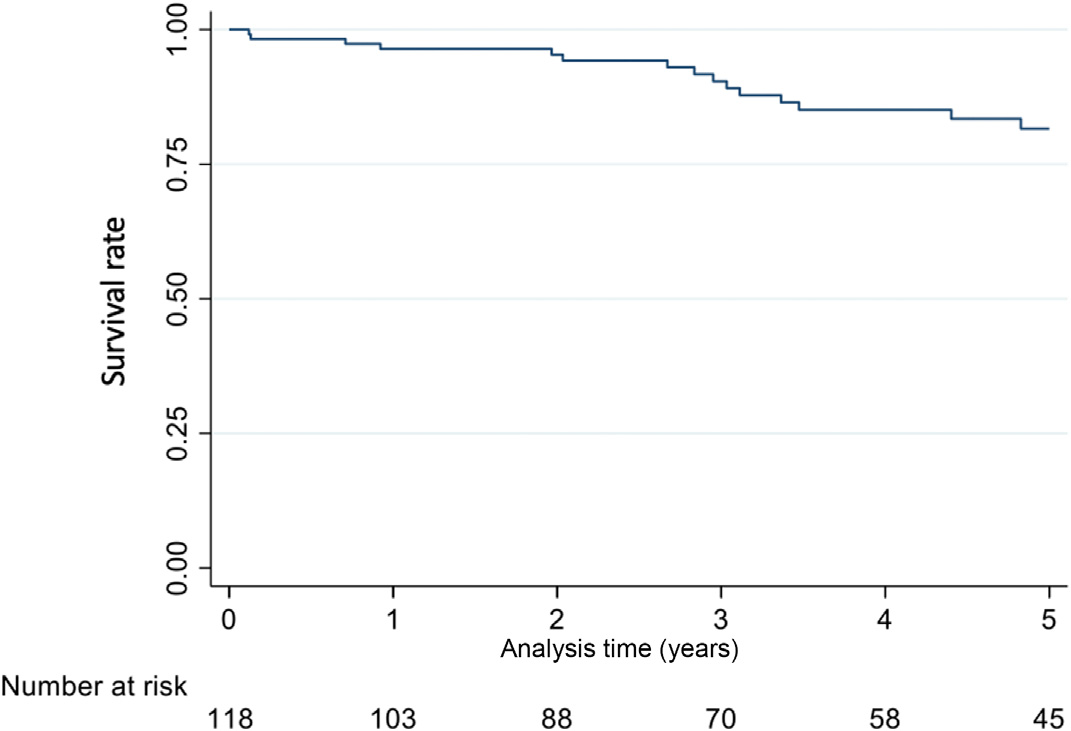
Overall Survival in Patients With Transthyretin Amyloid Cardiomyopathy Without Heart Failure Symptoms Overall survival in 118 patients with transthyretin amyloid cardiomyopathy without heart failure symptoms at initial evaluation was 96.5% (95% CI: 91%-99%), 90.4% (95% CI: 82%-95%), and 82% (95% CI: 71%-89%) at 1, 3, and 5 years, respectively.

Thirty-two patients died during follow-up. Sudden cardiac death was the cause of death in 4 patients, 2 died of terminal HF, and 2 died of other CV causes. In 11 patients (all with ATTR-v) the causes of death were non-CV, and in 13 (41%) the causes of death were unknown. Overall survival of the cohort was 96.5% (95% CI: 91%-99%), 90.4% (95% CI: 82%-95%), and 82% (95% CI: 71%-89%) at 1, 3, and 5 years, respectively (Figure 2). The breakdown of survival rates in patients with ATTR-v vs ATTR-wt was as follows: at 1 year, 97% (95% CI: 88%-99%) vs 96% (95% CI: 84%-99%); at 3 years, 90% (95% CI: 79%-95%) vs 91% (95% CI: 73%-97%); and at 5 years, 81% (95% CI: 68%-89%) vs 82% (95% CI: 52%-94%).

Additionally, 20 patients required permanent pacemakers during follow-up (10 patients for complete atrioventricular block, 1 after atrioventricular node ablation for heart rate control in AF, and 1 because of sinus node dysfunction. Indication was not available in 8 cases), 13 patients developed AF, and 1 patient experienced a stroke.

### ATTR-specific therapy in patients with ATTR-CM without HF

Fifty-six patients received pharmacologic ATTR-specific therapies during follow-up. The majority (n = 53 [91.4%]) were treated with TTR stabilizers (13 received diflunisal, 31 received tafamidis, and 9 received both). A smaller proportion (n = 13) were treated with genetic silencers. Of note, 2 patients underwent liver transplantation (Table 1).

Clinical, echocardiographic and electrocardiographic findings at baseline and at last follow-up of patients treated or not with stabilizers are shown in Table 3. Two patients who received TTR stabilizers and also underwent liver transplantation were excluded from the analysis. Similarly, 3 patients who did not receive stabilizers and received gene silencers (1 receiving it at baseline and 2 who started it during the first year of follow-up) were not included in the nonstabilizer group. Interestingly, the characteristics were similar in both groups except for an increased number of patients with ATTR-v and patients with higher eGFRs in the stabilizer-treated group (Table 3).

**Table 3:** Characteristics of Patients With ATTR-CM Treated With and Without TTR Stabilizers (N = 113)

	ATTR-CM With Stabilizer (n = 51)	ATTR-CM Without Stabilizer (n = 62)
Baseline characteristics		
Male	44	46
White (n = 111)	49	57
Median age at diagnosis, y	65 (54.5-71.5)	67 (53.5-78)
ATTR-v	34	29
ATTR-wt	17	33
Type of diagnosis		
Histological EMB	9	28
Histological extracardiac	23	3
Noninvasive	19	31
Era diagnosed		
Early (1993-1999)	0	8
Middle (2000-2009)	5	15
Late (2010-2019)	46	39
Circumstances leading to diagnosis (n = 108)		
Differential diagnosis LVH	9	8
Cardiac surveillance of ATTR-v	14	16
ATTR-v family screening	14	7
Incidental by scintigraphy	5	12
Conduction/rhythm abnormalities	4	4
Incidental by histology	3	2
Aortic stenosis workup	0	1
Others	0	9
Baseline clinical characteristics		
Sensorimotor polyneuropathy	30	40
History of embolism	0	2
History of AF	6	9
Hypertension	20	21
Coronary artery disease	6	6
Left-sided valve disease (at least moderate)	1	6
Pacemaker	4	1
Chronic kidney disease	1	2
Baseline blood test		
eGFR, mL/min/1.73 m^2^ (n = 99)	91.4 ± 24.9	74.4 ± 22.9
NT-proBNP, pg/mL (n =70)	338 (182.5-486)	230 (167.5-421.5)
Elevated troponin levels (n = 58)	10	4
Baseline ECG		
AF or atrial flutter	2	4
Conduction abnormalities	21	30
Baseline echocardiography		
Interventricular wall thickness, mm	15.2 ± 2.7	15.8 ± 3.4
Posterior wall thickness, mm (n = 111)	13.6 ± 2.4	14.3 ± 3
End-diastolic left ventricular diameter, mm (n = 107)	44.3 ± 4.1	44.7 ± 7.9
Left ventricular ejection fraction, % (n = 112)	60.5 ± 7.7	60.8 ± 11.8
Global longitudinal strain, % (n = 45)	−15.4 ± 3.2	−16.7 ± 1.5
Left atrial diameter, mm (n = 91)	39.5 ± 5.8	41.2 ± 6.6
Cardiac uptake at bone scintigraphy (n = 59)		
Grade 0	0	1
Grade 1	2	1
Grade 2	7	16
Grade 3	7	25
Follow-up		
Median follow-up, y	3 (1-6)	3 (1.3-6)
NYHA functional class at last contact (n = 109)		
I	37	36
II	8	15
III	4	6
IV	0	3
Laboratory values at last follow-up		
eGFR, mL/min/1.73 m^2^) (n = 79)	80.6 ± 23.5	64.7 ± 24.8
NT-proBNP, pg/mL (n = 60)	426.5 (204.3-960.3)	575.5 (178-1132.3)
ECG at last follow-up		
Sinus rhythm (n = 94)	35	34
Conduction abnormalities (n = 94)	14	19
Echocardiography at last follow-up		
Interventricular wall thickness, mm (n = 98)	16.1 ± 2.8	16.4 ± 3.4
Posterior wall thickness, mm (n = 90)	14.4 ± 2.7	15.2 ± 2.9
End-diastolic left ventricular diameter, mm (n = 72)	44.8 ± 4.5	45.1 ± 5.9
Left ventricular ejection fraction, % (n = 100)	58.4 ± 9.8	58.6 ± 11.5
Global longitudinal strain, % (n = 43)	−15.1 ± 3	−15.5 ± 2.7
Left atrial diameter, mm (n = 47)	40 ± 5.7	39.4 ± 5.3
Events		
AF during follow-up (n = 101)	6	7
Pacemaker implantation (n = 107)	13	6
Stroke (n = 106)	1	0
HF	13	24
Cardiac transplantation (n = 112)	0	2
Mortality	5	26

Values are n, median (IQR), or mean ± SD. Two patients who underwent liver transplantation and 3 who received gene silencers were excluded from the ATTR-CM with stabilizer and without stabilizer groups, respectively.

ATTR-CM = transthyretin amyloid cardiomyopathy; other abbreviations as in Tables 1 and 2.

Excluding the 2 patients who underwent liver transplantation, median time from diagnosis to initiation of stabilizers was 5 months (IQR: 0-16.5 months), with 18 patients being treated at baseline and 13 beginning therapy within the first year after initial evaluation.

**Figure 3:**
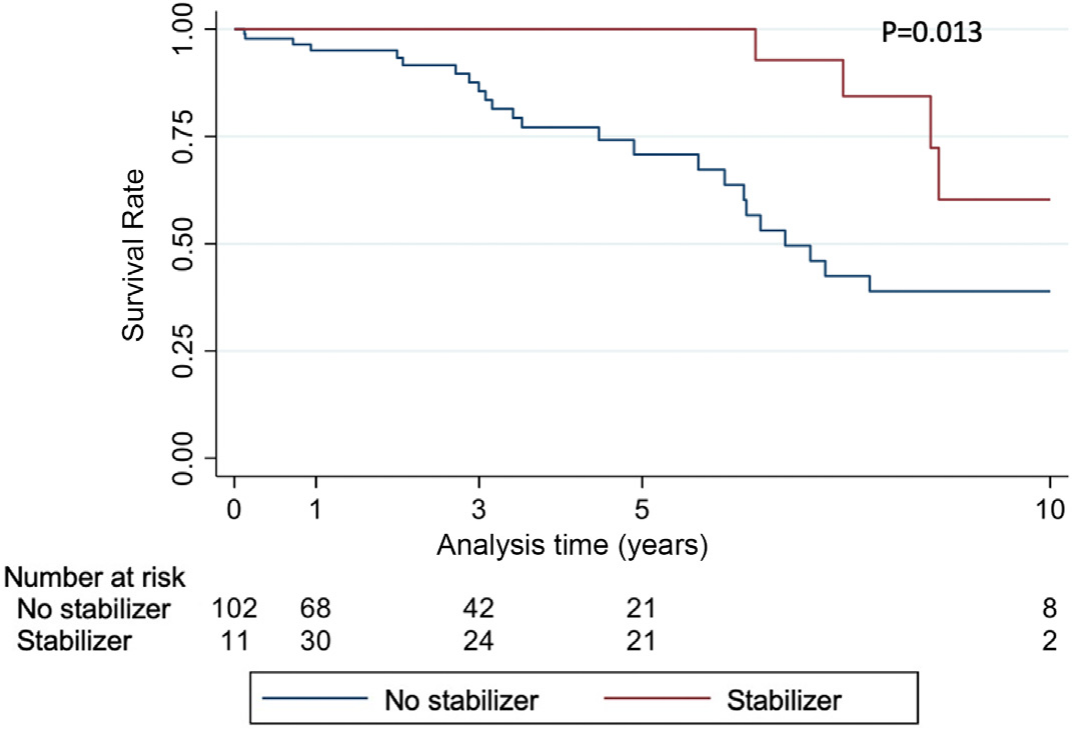
Survival in Patients With Transthyretin Amyloid Cardiomyopathy According to Treatment Unadjusted model showing survival in patients with transthyretin amyloid cardiomyopathy without heart failure symptoms treated with **(red line)** and without **(blue line)** transthyretin stabilizers.

**Figure 4:**
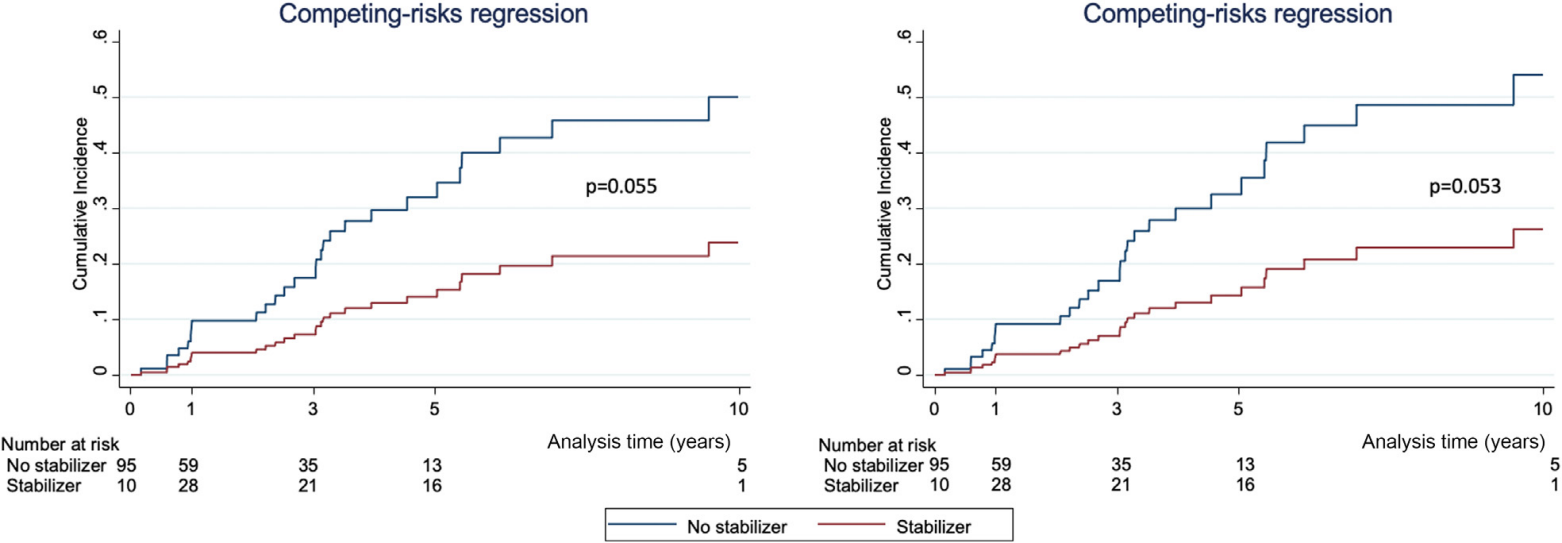
Time to Heart Failure Onset According to Treatment With Stabilizers Time to heart failure onset in patients with transthyretin amyloid cardiomyopathy without heart failure symptoms treated with and without transthyretin stabilizers: unadjusted **(left)** and adjusted **(right)** models.

Treatment with stabilizers was associated with improved survival both in unadjusted analysis (HR: 0.31; 95% CI: 0.12-0.82; *P* = 0.019) and after adjusting for sex, age, ATTR-CM type, and eGFR (HR: 0.18; 95% CI: 0.06-0.55; *P* = 0.002) (Table 4, Figure 3). In the time-to-HF analysis, treatment with stabilizers tended toward improved prognosis in the unadjusted analysis (SHR: 0.39; 95% CI: 0.15-1.02; *P* = 0.055), and this association remained of borderline statistical significance after adjusting for sex, age, ATTR-CM type, and eGFR (SHR: 0.39; 95% CI: 0.15-1.01; *P* = 0.053) (Table 4, Figure 4). Sensitivity analysis with bootstrapping provided an SHR of 0.39 (95% CI: 0.00-7,843.1; *P* = 0.854) in the unadjusted analysis and an SHR of 0.39 (95% CI 0.12-1.30; *P* = 0.125) in the adjusted model.

**Table 4:** Analysis of the Effect of Treatment With Stabilizers on Survival and HF Onset

	HR	95% CI	*P* Value
Unadjusted analysis			
Survival (n = 113)			
Treatment with stabilizers	0.31	0.12-0.82	0.019
HF onset (n = 105)			
Treatment with stabilizers	0.39^a^	0.15-1.02	0.055
Adjusted analysis			
Survival (n = 113)			
Treatment with stabilizers	0.18	0.06-0.55	0.002
Age	1.05	1.02-1.09	0.001
Female sex	0.93	0.41-2.11	0.86
ATTR-wt	0.16	0.05-0.50	0.002
eGFR	0.99	0.98-1.01	0.27
HF onset (n = 105)			
Treatment with stabilizers	0.39^a^	0.15-1.01	0.053
Age	1.02^a^	0.99-1.05	0.28
Female sex	0.73^a^	0.30-1.82	0.50
ATTR-wt	1.39^a^	0.61-3.17	0.44
eGFR	1.00^a^	0.99-1.02	0.72

Abbreviations as in Tables 1 and 2.

a Subdistribution HR.

## Discussion

This multicenter study presents, for the first time to our knowledge, data on patients with ATTR-CM without HF symptoms at diagnosis. We show that one-third of these patients developed HF during a median follow-up period of 3.7 years and that a substantial number of patients developed other CV complications. Moreover, our data show that patients treated with TTR stabilizers can potentially benefit from early treatment (Central Illustration).

**Central Illustration:**
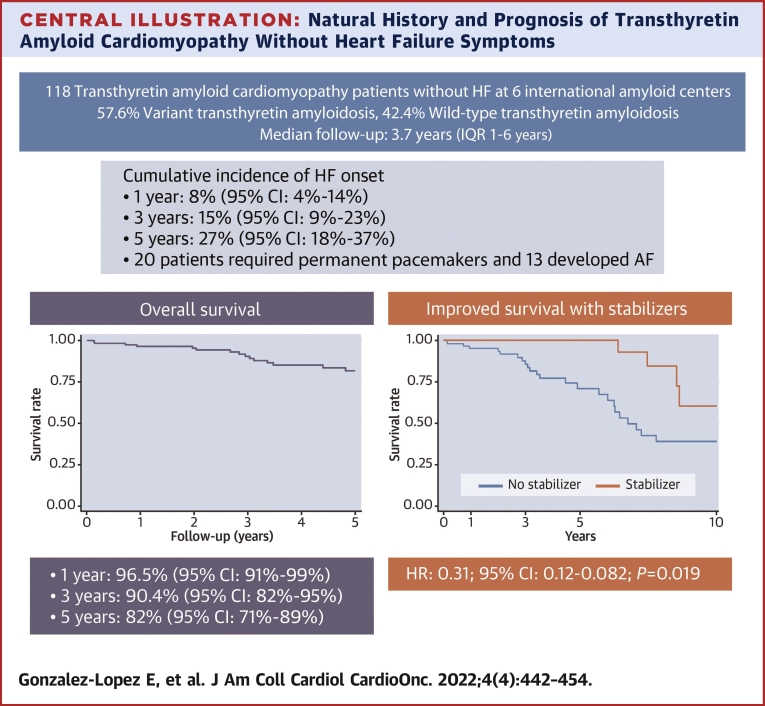
Natural History and Prognosis of Transthyretin Amyloid Cardiomyopathy Without Heart Failure Symptoms Analysis of a cohort of 118 patients with transthyretin amyloid cardiomyopathy recruited from 6 international amyloid centers showed that after a median follow-up period of 3.7 years, 32% patients developed heart failure (HF) symptoms, with a cumulative incidence of HF onset at 1, 3, and 5 years of 8% (95% CI: 4%-14%), 15% (95% CI: 9%-23%), and 27% (95% CI: 18%-37%), respectively. Treatment with transthyretin stabilizers was associated with improved survival. ATTR-CM = transthyretin amyloid cardiomyopathy; ATTRv = variant transthyretin amyloidosis; ATTRwt = wild-type transthyretin amyloidosis; PPM = permanent pacemakers.

### Clinical characterization and prognosis of patients with ATTR-CM without HF

Once thought to be a rare cardiac disease, ATTR-CM is now recognized as being much more frequent and contributes to common cardiac presentations such as HF with preserved ejection fraction, degenerative aortic stenosis, or hypertrophic cardiomyopathy in a substantial number of patients.^7^, ^8^, ^9^^,^^16^^,^^17^ Recognition of the contribution of ATTR-CM in these clinical settings combined with advances in noninvasive cardiac imaging and the emergence of new effective specific therapies have led to a substantial increase in the number of diagnosed patients with ATTR-CM.^5^^,^^6^ Moreover, the number of patients diagnosed incidentally or at very early stages has also risen considerably in recent years.

To our knowledge, there has been no systematic analysis of cardiac progression among patients with ATTR-CM without HF symptoms, which makes patient counseling difficult in this growing population. Available prognostic information in ATTR-CM is dominated by series of patients with advanced HF and severe cardiomyopathy because, until recently, ATTR-CM was mainly recognized only in individuals with advanced disease.^10^, ^11^, ^12^

We present the prognostic data of a multicenter cohort of patients with ATTR-CM without HF symptoms at baseline evaluation. Although exercise testing was not required to confirm the absence of symptoms, the low median NT-proBNP concentration of the cohort supports its early stage, particularly considering the advanced median age of the population and the presence of baseline AF and chronic kidney disease in a non-negligible number of subjects. In our study, patients were diagnosed in a variety of ways, including during ATTR-v surveillance but also incidentally on scintigraphy or biopsy.

We show that progression to clinical HF occurs in a remarkable proportion (one-third) of these patients in a relatively short period of time. Moreover, AF and conduction abnormalities appeared frequently, and nearly 20% of patients from the overall cohort required pacemaker implantation during follow-up.

Our findings should help when counseling patients with asymptomatic ATTR-CM, as the closest available data come from patients with ATTR-CM with the less severe stage of disease (stage I) from the 2 ATTR-CM prognostic scores available (the Mayo Clinic and UK National Amyloid Center [NAC] scores).^11^^,^^12^ Patients with stage I ATTR-CM by both staging scores exhibited NT-proBNP levels <3,000 pg/mL, with those in Mayo Clinic stage I showing troponin T values <50 ng/L and those in NAC stage I with eGFR >45 mL/min. Not unexpectedly, the stage I groups of both scores do not reflect well the prognosis of ATTR-CM without HF symptoms as assessed in our study. The median overall survival described for stage I patients was 66 and 69 months for the Mayo Clinic and NAC scores, respectively.^11^^,^^12^ In contrast, survival of patients with ATTR-CM without HF symptoms does not seem to be so severely impaired, with 90% and 82% survival at 3 and 5 years, respectively.

The superior survival observed in our cohort is in line with data from Columbia University showing better prognosis of ATTR-CM in patients with NYHA functional class I and those not receiving diuretic agents.^18^

### ATTR-specific therapies

ATTR-CM progression is characterized by loss of cardiac performance, worsening of cardiac biomarkers, decline in functional capacity, recurrent hospitalizations, disability, and ultimately death, which is usually CV in nature and due to underlying disease.^5^^,^^6^^,^^19^

Access to novel, effective, and specific compounds for both ATTR-wt and ATTR-v has recently grown and has the potential to change the natural course of the disease. Therapies that reduce the production of mutated TTR or stabilize circulating TTR molecules, preventing their dissociation into monomers, have already shown efficacy against ATTR-v with polyneuropathy, and tafamidis, a TTR stabilizer, has proved effective in reducing mortality and CV-related hospitalization in patients with ATTR-CM, resulting in its approval for ATTR-CM by the U.S. Food and Drug Administration, European Medicines Agency, and other regulatory agencies.^13^^,^^14^ Following in the steps of tafamidis, gene-silencing molecules (patisiran, vutrisiran, and eplontersen) and a second stabilizer (acoramidis) are currently being tested in randomized controlled trials for ATTR-CM (NCT03997383, NCT04153149, NCT04136171, and NCT03860935).

Although early diagnosis of a progressive fatal disease could potentially offer a number of therapeutic opportunities, the tafamidis ATTR-CM trial and all the ongoing ATTR-CM trials included only patients with ATTR-CM with HF symptoms.

To complicate things further, ATTR-CM commonly affects elderly individuals, who could be affected by many other diseases, which might influence decisions about the initiation of long-term and expensive treatments, despite the fact that the Food and Drug Administration and European Medicines Agency have authorized tafamidis for the treatment of ATTR-CM without restricting it to patients with overt HF.^20^

Given the substantial number of events found during follow-up among patients with ATTR-CM without HF symptoms in our study and the underlying pathophysiological mechanism of the disease, early modification of its natural history by specific TTR therapies could be meaningful. To gain insight into this critical question, we analyzed the HF and mortality outcomes in patients with ATTR-CM without HF symptoms. We found that patients who were treated with stabilizers had improved survival and tended toward delayed progression to clinical HF despite the observational and retrospective nature of the present study and the potential of confounders such as advanced neurologic stage or other comorbidities. Indeed, the reported survival benefit of tafamidis from clinical trial data was observed after 18 months of treatment, unlike in our analysis, in which it seemed to occur earlier, suggesting that other factors might have played a role in the survival differences observed between ATTR-CM patients without HF who received stabilizers and those who did not. Nevertheless, our results are in line with the better outcomes reported in less severely affected patients in both the ATTR-CM tafamidis trial and polyneuropathy trials of ATTR-v genetic silencers.^13^^,^^14^

Although our findings would ideally need to be confirmed in randomized studies, our results provide support to consider early initiation of stabilizing agents in asymptomatic patients with ATTR-CM.

### Study limitations

Important limitations to this investigation are worth noting. This was a retrospective study, not a randomized control trial. Although clinical characteristics (except proportion of patients with ATTR-v and eGFR) and echocardiographic and electrocardiographic parameters did not differ between patients who received TTR stabilizers and those who did not, we cannot exclude a potential selection bias for patients having a less severe disease phenotype. The number of patients analyzed was limited, and therefore results should be interpreted with caution. Moreover, a substantial proportion of patients were diagnosed following family screening or ATTR polyneuropathy assessment. Therefore, our findings may not be generalizable, as disease processes may progress differently on the basis of mode of diagnosis and ATTR variant. Furthermore, patient information was collected across a prolonged time interval, and not all parameters were available in all patients. It should be noted that date of HF onset was not available in 8 patients who developed HF during follow-up and had to be excluded from time-to-HF analyses, potentially influencing the results. However, the clinical characteristics of these 8 individuals did not differ from those of the overall cohort for whom HF date was available. Also, participating centers are highly specialized amyloidosis centers, and therefore, referral and survival bias cannot be excluded. Last, time to HF development was assessed from baseline evaluation at participating centers to HF presentation and not from diagnosis, implying an indisputable lead-time bias.

## Conclusions

Almost one-third of patients with ATTR-CM without HF symptoms at initial evaluation developed HF after a median follow-up period of 3.7 years. The rates of other events, namely, pacemaker implantation and AF, were also remarkable. Treatment with TTR stabilizers was associated with less progression to clinical HF and improved survival. Further studies are necessary to clarify the most appropriate therapeutic approach for patients with asymptomatic ATTR-CM, but our results provide support to consider initiating stabilizing agents at early stages of the disease.

Perspectives **COMPETENCY IN MEDICAL KNOWLEDGE:** ATTR-CM patients without HF symptoms have poor prognosis with one in three developing HF symptoms during a median follow-up of 3.7 years and a substantial number experiencing other CV complications.

**TRANSLATIONAL OUTLOOK:** Additional studies should define the most appropriate group of asymptomatic patients to screen for ATTR-CM. Further studies are needed to define the benefit of ATTR-specific therapies in asymptomatic ATTR-CM patients.

## Funding Support and Author Disclosures

This work was supported by grants from Instituto de Salud Carlos III (PI18/0765 and PI20/01379). Dr Gonzalez-Lopez has received speaker fees from Pfizer and Alnylam; has received consulting fees from Pfizer and Proclara; and has received research and educational support to her institution from Pfizer, BridgeBio, and Alnylam. Dr Obici has received speaker and consulting fees from Pfizer, Alnylam, and Akcea. Dr AbouEzzeddine has received research grant support from Pfizer. Dr Mussinelli has received speaker fees from Pfizer and Akcea. Dr Dispenzieri has received consulting fees from Janssen and Akcea; and has received research support from Pfizer, Alnylam, Celgene, and Takeda. Dr Perlini has received speaker and consulting fees from Pfizer, Alnylam, and Akcea. Dr Palladini has received speaker fees from Janssen-Cilag, Pfizer, and Siemens; and has participated on an advisory board for Janssen Cilag. Dr Damy has received research grants or consulting fees from Alnylam, Akcea, Pfizer, and Prothena. Dr Grogan has received research grant support and consulting fees to her institution from Alnylam, Eidos, Pfizer, and Prothena. Dr Maurer has received grant support from National Institutes of Health (R01HL139671-01, R21AG058348, and K24AG036778); has received consulting income from Pfizer, GlaxoSmithKline, Eidos, Prothena, Akcea, and Alnylam; and has received clinical trial funding to his institution from Pfizer, Prothena, Eidos, and Alnylam. Dr Garcia-Pavia has received speaker fees from Pfizer, BridgeBio, Alnylam, and Ionis; has received consulting fees from Pfizer, BridgeBio, AstraZeneca, NovoNordisk, Neuroimmune, Alnylam, Alexion, and Attralus; and has received research and educational support to his institution from Pfizer, BridgeBio, and Alnylam. All other authors have reported that they have no relationships relevant to the contents of this paper to disclose.
